# Characterizing Focused Attention and Working Memory Using EEG

**DOI:** 10.3390/s18113743

**Published:** 2018-11-02

**Authors:** Zainab Mohamed, Mohamed El Halaby, Tamer Said, Doaa Shawky, Ashraf Badawi

**Affiliations:** 1 Center for Learning Technologies, University of Science and Technology, Zewail City, Giza 12578, Egypt; zrajab@zewailcity.edu.eg (Z.M.); tsaid@zewailcity.edu.eg (T.S.); abadawi@zewailcity.edu.eg (A.B.); 2 Mathematics Department, Faculty of Science, Cairo University, Giza 12613, Egypt; halaby@sci.cu.edu.eg; 3 Engineering Mathematics Department, Faculty of Engineering, Cairo University, Giza 12613, Egypt

**Keywords:** cognitive skills measurement, electroencephalography, short-time fourier transform, classification

## Abstract

Detecting the cognitive profiles of learners is an important step towards personalized and adaptive learning. Electroencephalograms (EEG) have been used to detect the subject’s emotional and cognitive states. In this paper, an approach for detecting two cognitive skills, focused attention and working memory, using EEG signals is proposed. The proposed approach consists of the following main steps: first, subjects undergo a scientifically-validated cognitive assessment test that stimulates and measures their full cognitive profile while putting on a 14-channel wearable EEG headset. Second, the scores of focused attention and working memory are extracted and encoded for a classification problem. Third, the collected EEG data are analyzed and a total of 280 time- and frequency-domain features are extracted. Fourth, several classifiers were trained to correctly classify and predict three levels (low, average, and high) of the two cognitive skills. The classification accuracies that were obtained on 86 subjects were 84% and 81% for the focused attention and working memory, respectively. In comparison with similar approaches, the obtained results indicate the generalizability and suitability of the proposed approach for the detection of these two skills. Thus, the presented approach can be used as a step towards adaptive learning where real-time adaptation is to be done according to the predicted levels of the measured cognitive skills.

## 1. Introduction

Detecting cognitive states and skills is an important step towards adaptive learning, in which the learning material and pace are adjusted to match some collected data about learners during a learning task. In addition to the invasive approach in which learners are asked to provide some information about their learning sessions, an automated non-invasive one is also possible. A promising methodology for the automated collection of data during a mental task includes the use of bio-sensors that could measure subjects’ emotions, attention, and engagement in a non-intrusive way in order to not interfere with her/his learning process.

In this paper, an approach for measuring two basic cognitive skills that affect the outcomes of the learning process using bio-signals is presented. The measured cognitive skills include focused attention (FA) and working memory (WM). FA means that all subjects’ activities involve active cognitive processes such as problem-solving and critical thinking [1]. WM, on the other hand, is a type of short-term memory that allows subjects to store and manipulate temporary information [2]. As presented in [3], the cognitive skills of learners in a learning task play a major role in determining how well they perceive the knowledge being presented.

One of the bio-signals that could be measured in a convenient, non-intrusive way is the brain activity. The electroencephalogram (EEG) is the brain electrical activity measured by mounting electrodes on the scalp, where the activity of millions of cortical neurons produce an electrical field that can be measured from the human scalp [4]. Analyzing EEG can be effectively used in measuring emotions and some other psychological states of subjects, especially after advancements in the technology and the availability of convenient wireless devices that can provide reasonable accuracy in measuring brain activity similar to that provided by expensive medical devices.

Utilization of several biosensors to detect various cognitive skills and the application in education is not a novel approach. Many studies have tackled the same problem with different tools and analysis techniques. In regard to attention, the authors in [5] have built a classification model that is able to classify the EEG signals of 24 participants to attentive and inattentive while listening to instructions. Support vector machines are used, and the best achieved accuracy is 76.8%. In another relevant study [6], the relation between changes in spectral analysis of EEG signals for 18 subjects and sustained visual attention is analyzed in a real classroom setting. Changes in power values of some frequency bands (alpha, theta, beta, and gamma [7]) are found to be features that can characterize sustained visual attention. Also in [8], four attention states have been classified using artificial neural networks with classification accuracies that range from 56.5–79.75%. Moving to working memory, the authors in [9] have built a support vector machines classifier that is able to discriminate between working memory and the recognition of cognitive states from EEG data with a classification accuracy of 79%. In addition, the work in [10] utilizes the characteristics of power values in different frequency bands for EEG data during working memory tasks that vary in type and complexity. Changes in theta to beta EEG frequency bands are found to indicate the performance of WM. Currently, there are two fundamental principles that might help in understanding brain-behavior relationships; namely, segregation and integration [11]. The former is based on the fact that cerebral cortex can be subdivided into distinct modules, each of them has its own structure and functionality. The latter, on the other hand, is based on the fact that no region of the brain can perform a certain task individually, instead, the interactions and exchange of information between different regions have to be done for any mental or motor functionality to be performed. The analysis conducted in this paper is based on the latter principle, where electric signals are measured from different locations on the scalp and the gathered information is integrated and analyzed. The presented approach includes the following main steps. First, the subjects put on a wireless headset that measures EEG signals from 14 different electrodes distributed across the scalp. Second, they undergo a scientifically-validated cognitive assessment test. This step helps elicit and stimulate the signals related to the cognitive skills that need to be measured. Third, the collected EEG signals are analyzed to find out whether there are relationships between the extracted EEG features and the measured cognitive skills.

The rest of the paper is organized as follows. In Section 2, the related work is presented. The details of the materials and methods are presented in Section 3. In Section 4, the results of the proposed approach are discussed. Finally, in Section 5, conclusions and directions for the future work are outlined.

## 2. Related Work

The literature includes many studies that have established EEG and event-related potentials (ERPs) as the primary tools available to scientists investigating neural indexes of cognition in different application areas [12]. Thus, there is a large number of studies that detect different cognitive states such as cognitive impairment, levels of alertness and drowsiness, attention, workload, and memory from EEG signals [13,14,15,16]. Most of the available approaches employ machine learning algorithms for building models that can predict cognitive states based on some extracted features from the EEG signals.

For instance, as an example of the analysis of EEG in medical applications to detect cognitive impairment, the authors in [17] presented a methodology based on EEG to detect Alzheimer’s disease and its prodromal form (mild cognitive impairment). 5-min of spontaneous EEG activity were recorded using a 19-channel EEG system at a sampling frequency of 200 Hz. Subjects were asked to stay in a relaxed state, awake, and with closed eyes during EEG acquisition. The features extracted include spectral and non-linear features including relative power in the conventional frequency bands, median frequency, individual alpha frequency, spectral entropy, central tendency measure, sample entropy, fuzzy entropy, and auto-mutual information. Finally, three classifiers were trained using a subset of the used features namely: linear discriminant analysis (LDA), quadratic discriminant analysis (QDA), and multi-layer perceptron artificial neural network (MLP). MLP showed the highest diagnostic performance in determining whether a subject is not healthy with sensitivity of 82.35% and positive predictive value of 84.85%. As another example, a model for deep learning, namely deep belief networks (DBN) was used in [18]. In this approach, DBNs were applied in a semi-supervised paradigm to model EEG waveforms for the classification and anomaly detection of EEG signals. EEG signals were recorded while 11 subjects received therapeutic hypothermia treatment comatose after cardiac arrest. Then, randomly subsampled 1000 2-min segments were extracted. The authors compared the use of raw, unprocessed data to hand-chosen features and found that DBN has comparable performance with support vector machines (SVM) and k-nearest neighbor (KNN) classifiers and has fast test time, even on high-dimensional raw data.

In a different application, EEG has been employed to detect neuro-activity related to encoding different movements, viewing different images, as well as encoding drivers’ cognitive states and alertness level. For instance, in [19], the main objective was to detect how the features extracted from EEG vary with four tasks: slow walking, navigating and counting, communicating with radio, and studying mission map. The experimental setting involves a user outfitted with wearable monitoring, communication, and mobile computing equipment walking outside. The monitoring equipment is a BioSemi Active Two EEG system with 32 electrodes. Vertical and horizontal eye movements and blinks were recorded with electrodes below and lateral to the left eye. Using the power spectral density of signals from seven different sites, the model classified. Classification rate was 83% using 14 features. Moreover, in [20], the authors showed how to design and train convolutional neural networks (ConvNets) to decode movement-related information from the raw EEG without handcrafted features and highlights the potential of deep ConvNets when combined with advanced visualization techniques for EEG-based brain mapping. In this study, three ConvNets with different architectures, with the number of convolutional layers ranging from 2 layers in a “shallow” ConvNet over a 5-layer deep ConvNet up to a 31-layer residual network (ResNet), were used. The authors evaluated a large number of ConvNet design choices and they showed that end-to-end deep ConvNets can reach accuracies at least in the same range as other tools for decoding movement-related information from EEG.

Also, in [21], an algorithm to decode brain activity associated with different types of images is proposed. In this algorithm, ConvNet is modified for the extraction of features. Then, a t-test is used for the selection of significant features and finally, likelihood ratio-based score fusion is used for the prediction of brain activity. Multivariate pattern analysis (MVPA) is used by encoding the complete input into ConvNet with likelihood ratio based on score fusion. Continuous EEG data were recorded with a 128 channel with a sampling frequency of 250 Hz. The data were pre-processed where the signals were filtered from 0.3 to 30 Hz using a bandpass filter. Eye-blink (EOG) artifacts were corrected using an adaptive artifact correction method. The proposed method has prediction accuracy of 79.9%. Moreover, in [22], the authors used a recurrent neural network (RNN) to decode the EEG data to help them find out whether EEG data can be used to accurately classify whether someone is viewing a 2D or 3D image. The collected data recordings from 12 human subjects. Each stimulus was presented for 1.65 s at a sampling rate of 256 Hz. However, the best obtained accuracy was 72% and the authors concluded that it is not straightforward to apply RNNs to EEG data.

As an example of how EEG was employed for the encoding of cognitive states of subjects in a learning-related application, in [23], an intelligent tutoring system (ITS) that can dynamically tailor its instructional strategies to impose an appropriate amount of cognitive load for each participant is presented. The authors showed the feasibility of collecting EEG data while students learn from an ITS using a hat-like headset. The models use EEG spectral features to differentiate between low and high cognitive load training tasks via partial least squares regression. Linear mixed effects regressions show that cognitive load during some of the tasks is related to performance on the questions. Also, in [24], the authors studied whether students’ neural reliability can be quantified in a real-time manner based on recordings of brain activity in a classroom setting using a low-cost, portable EEG system. Video stimuli is presented while recording EEG activity as the subjects watched short video clips of approximately 6 min duration, either individually or in a group setting. Inter-subject correlation (ISC) is used as a marker of engagement of conscious processing. To measure the reliability of EEG responses, correlated components analysis is used to extract maximally correlated time series with shared spatial projection across repeated views within the same subject (inter-viewing correlation, IVC), or between subjects (inter-subject correlation, ISC). Similarity of responses between subjects is detected which showed the reliability of ISC as a measure for engagement. Moreover, in [25], the authors developed a feature extraction scheme for the classification of EEG signals during a complex cognitive task, and in rest condition with the eyes open. The discrete wavelet transform is applied on EEG signals and the relative wavelet energy is calculated in terms of detailed coefficients (and the approximation coefficients of the last decomposition level). The extracted relative wavelet energy features are passed to classifiers for the classification purpose. The performances of four different classifiers (KNN, SVM, MLP, and Naive Bayes) were evaluated. Some classifiers, including SVM and MLP, achieved over 98% accuracy in this task. However, a very small number of participants was included in this study (only 8 participants were included). Also, in [26], EEG signals were used to assess cognitive load in multimedia learning tasks. The EEG signals were recorded from 34 subjects using 128-channel device at a sampling frequency of 250 Hz. In addition, some other bio-signals were recorded to help pre-process EEG signals, including eye blinks and heart rate. The learning tasks included multimedia animations based on plant anatomy. Then, a memory recall test was given to the subjects. Four distinct frequency bands were used to extract the three spectral features based on entropy features. Partial directed coherence (PDC) was also applied to determine the connectivity patterns among the selected channels of the brain. Three types of classifiers (Naïve Bayes, SVM with linear kernel, and SVM with radial basis function kernel) were used to classify the extracted features. The best classification accuracies were obtained using the features of the alpha waves. In addition, in [27], an approach to measure working memory (WM) across tasks is provided. The WM tasks used included verbal n-back, spatial n-back [28] and the multi-attribute task battery [29]. EEG acquisition was performed in a magnetically and electrically-shielded room using 30-channel EEG data. Then, a power spectral density (PSD) estimator using Burg’s method was used to calculate the sums of PSD in 7 frequency bands, which resulted in 7 features of each of the 30 channels. The support vector machine regression algorithm (SVMR) with a linear kernel function was used. Although some satisfactory results were found for some subjects in cross-task regression, however, as reported in the study, overall results were not satisfactory.

## 3. Materials and Methods

### 3.1. Electroencephalogram (EEG) Data Acquisition

**Figure 1:**
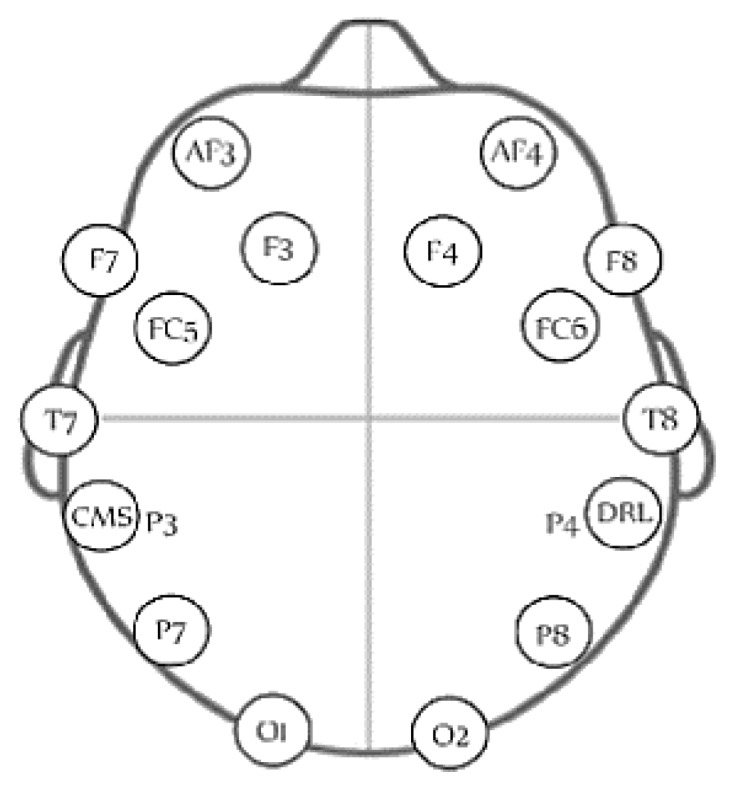
The distribution of the 14 electrodes of the used EEG headset [30].

The EEG signals are measured using the wireless headset (Emotiv Epoc, San Francisco, CA, USA). The headset has 16 channels, where 14 of them are used for collecting the EEG signals from different parts of the scalp and two are used as reference points. The sensors measure EEG data from AF3, AF4, F3, F4, FC5, FC6, F7, F8, T7, T8, P7, P8, O1, O2 based on the 10–20 system as shown in Figure 1. EEG signals are sampled at 128 samples/s.

### 3.2. Experimental Setup

#### 3.2.1. Target Population

The population includes all the students in the university who meet the inclusion criteria and were available for the actual experiments. Equal opportunity was given to all students to participate. The exclusion criteria were based on the following. Any student with neurological disorder (epilepsy, Alzheimer disease and other dementias, cerebrovascular diseases including stroke, migraine and other headache disorders, brain tumors, traumatic disorders of the nervous system due to head trauma, and neurological disorders as a result of malnutrition) would be excluded. Also, the students who suffered from any type of skin allergy and those who took any medications that might interfere with the physiological data were excluded. To recruit the subjects, an email was sent to all students, informing them about the experiment. The email contained information about the purpose of the study, the experimental procedure, discomforts that the subjects might suffer from while putting on the headset, confidentiality of the study, instructions to be done on the day of the experiment, and the incentives that they would receive as a sign of appreciation for their contribution. In addition, a consent form was signed indicating that the subjects satisfy the inclusion criteria in addition to the aforementioned information prior to the experiment. In total, the experiment included 86 subjects (72 males, 14 females) with ages ranging from 18 to 23 years (µ = 19.76, σ = 0.951). The study was approved by the Research Office in Zewail City.

#### 3.2.2. Procedure and Task Description

In order to record the EEG signals associated with different cognitive skills, the subjects undergo a scientifically-validated cognitive assessment battery while putting on the EEG headset. The test is available at [31]. The test consists of a series of fixed activities that stimulate perception, memory, attention and other cognitive states. The main reason for choosing this test is that it is clinically-proven; in addition, it has been used in several scientific publications [32,33,34,35]. The outcome of interest represents the given test scores or measures of the cognitive skills for each subject. The cognitive skills measured include 23 basic skills and 5 compound skills. The five compound skills include memory, attention, coordination, perception, and reasoning. Each of these 5 compound skills are composed of a set of basic skills. For instance, the score of perception is the average of the scores of six basic skills; namely recognition, auditory perception, spatial perception, visual scanning, visual perception, and estimation. On the other hand, focused attention (FA) and working memory (WM) belong to the basic skills that are directly measured and whose scores are directly reflected in the test. The numeric scores given to cognitive skills vary from 0 to 800 with a categorization of the profile to low (score<200), moderate (200≤score<400) and high (score≥400). Thus, in the conducted experiments, FA and WM, scores were encoded to these three levels.

**Figure 2:**
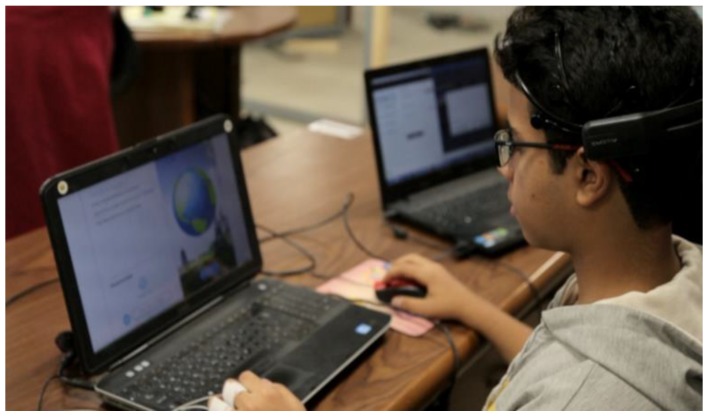
Experimental set-up. A subject undergoes the cognitive assessment test while wearing the EEG headset.

The data collection was extended across three months, where three subjects, on average, were included each day. The reason for this is that we need to clean the contacts of the headset to make sure that the signals received from them were as strong as possible. Each session lasted for an average of 40 min including the instructions given to the participant and the setup time. Figure 2 shows the experimental setup.

It should be mentioned that the quality of the measured signals varied from one subject to another due to the differences in scalp and hair thickness. More specifically, it varied from 17% to 85%, where the lowest contact quality was obtained for female participants with thick and long hair.

### 3.3. Artifact Identification and Removal

EEG signals have very small amplitudes (approximately from 40 to 100 µV), hence they easily get buried in larger interfering signals that stem from the electrochemical activity of other cells and their ability to conduct electrical current. These signals are called artifacts and they should be removed from the EEG signal before any analyses could be done. Artifacts can be divided into external and internal artifacts [4]. External artifacts are caused by outer actions, such as interference with the power line signals or electronic devices. Internal artifacts, on the other hand, are related to the actions made by the subjects. Those include body movements, movement of eye ball (oculogenic potentials), and cardiac activity.

There are several methods for detecting and removing artifacts from EEG signals. The application of digital filters, statistical analysis, autoregression modeling, and independent component analysis are among the most used methods. However, the effective utilization of these methods requires prior knowledge about where the signal is and where the noise comes from.

In the conducted experiments, only the basic preprocessing is performed. The reason for that is twofold. First, no prior knowledge about where the signal might be is available. Second, any excessive preprocessing actually distorts the signal and might mislead the analyses.

The high-frequency external artifacts were originally removed by the 50 Hz low pass filter included in the headset. This filter affects frequencies from 43 Hz and above. Thus, the maximum frequency to be analyzed was 43 Hz. Additionally, the DC offset (mean amplitude displacement from zero) which was added to the signal during sampling was removed using a first order finite impulse response (FIR) filter with cut-off frequency of 0.16 Hz, which resulted in a preprocessed signal with no DC component (*NoDC*) that is suitable for frequency analysis. Finally, some statistical analyses were performed to detect the outliers, i.e., the signals with exceptionally large amplitudes (greater than 3× mean value). Since the data collection sessions were relatively long, those samples were removed. This resulted in a further preprocessed signal, denoted by *NoOutlier*. The percentage of removed samples as a result of the performed preprocessing varied from one subject to another with values of 2%, 15%, 0.3%, and 0.11%, for the maximum, minimum, mean, and standard deviation, respectively.

### 3.4. Feature Extraction

#### 3.4.1. Time-Domain Features

In the signal’s time domain, the extracted features are primarily statistical ones. The extracted time-domain features include the following:

1. Minimum
2. Maximum
3. Mean
4. Variance
5. Standard Deviation
6. Coefficient of Variance
7. Kurtosis
8. Skewness
9. 25 Quartile
10. 50 Quartile
11. 75 Quartile
12. Shapiro–Wilk test (test statistic and *p*-value)
13. Hjorth mobility and complexity parameters

Each of the above features is calculated for each of the 14 channels. In addition, the Hjorth mobility and complexity parameters are calculated as given by Equations (1) and (2) [36], respectively:

(1) $$Mobility=\sqrt{\frac{var\left(\frac{dx\left(t\right)}{dt}\right)}{var\left(x\left(t\right)\right)}}$$

(2) $$Complexity=\frac{Mobility\left(\frac{dx\left(t\right)}{dt}\right)}{Mobility\left(x\left(t\right)\right)}$$

where x(t) is the signal, var is its variance, and the forward difference formula is used for the calculation of $\frac{dx\left(t\right)}{dt}$.

#### 3.4.2. Frequency Domain Features

There are variations in the EEG signals that cannot be shown only in the time domain. Thus, we transformed the collected EEG signals to the frequency (spectral) domain to be able to extract such variations. Short-time Fourier transform (STFT) is used for transforming EEG signals to the spectral domain. This is done by the calculation of fast Fourier transform (FFT) on short segments or windows of fixed length on the analyzed signal. FFT is a time-efficient numerical implementation of the Fourier transform (FT). For a given time-series signal *x*[*n*] with *N* sample points (*n* = 1, 2… *N*), FFT is given by Equation (3).

(3) $$fft\left(\omega \right)=\sum_{n=1}^{N}x\left[n\right]e^{-i\omega n},\;\omega =\frac{2\pi m}{N},\;0\leq m\leq N-1$$

where *ω* = 2π*f*/*fs* represents the angular frequency discretized in *N* samples and fs is the sampling frequency [37]. STFT is an extension of the Fourier transform that addresses two main limitations of the FT for EEG time-frequency analyses. First, FT obscures time-varying changes in the frequency structure of the data. Second, FT assumes that the data are stationary, which is not true for EEG signals. To address this, data in short segments have to be tapered, which attenuates the amplitude of the data at the beginning and the end of the time segment. This is important for preventing edge artifacts from contaminating the time-frequency results. However, tapering attenuates valid EEG signal, thus overlapping time segments have to be used. In the conducted analysis, a Hann window is used with 50% overlapping. After transforming the signal to the frequency domain using STFT, Power spectral density (PSD) is calculated. PSD is an important feature of the signal in the frequency domain that represents the contribution of each individual frequency component to the power of the whole signal segment. PSD can be estimated using periodogram [38], which can be calculated as given in Equation (4).

(4) $$per\left(\omega \right)=\frac{1}{N}{\left|fft\left(\omega \right)\right|}^{2}$$

Using a normalized periodogram, the relative contribution of each frequency component to the total power can be estimated as given by Equation (5).

(5) $$per_{norm}=\frac{1}{N}{\left|fft\left(\omega \right)\right|}^{2}/\overset{2\pi \left(N-1\right)/N}{\underset{\omega =0}{\sum }}per\left(\omega \right)$$

As suggested in cognitive physiology, the power of four main frequency bands in the EEG signals can reflect cognitive activity in the brain. These frequency bands include theta (4–8 Hz), alpha (8–12 Hz), beta (12–30 Hz), and gamma (30–60 Hz). Thus, the relative power of the four frequency sub-bands are calculated and used as features for the EEG signals in the frequency domain. In addition, the total average power in each channel is calculated.

It should be mentioned that for many signals, it is difficult to interpret raw power data at different frequency ranges. This is mainly because the frequency spectrum tends to show decreasing power at increasing frequencies; a phenomena that is called the 1/f power scaling [39]. Thus, the power at higher frequencies (e.g., gamma) has much smaller magnitude than the power at lower frequencies (e.g., theta). In addition, it is not possible to aggregate raw power values across subjects because of the individual differences, which affects the power values (e.g., hair and skull thickness). The 1/f power scaling also affects task-related power, especially in the frequencies that tend to have high power during baseline or resting period. To address these limitations, usually baseline normalization is performed. In this paper, decibel (dB) conversion is used, which is one of the most commonly used approaches for baseline normalizations in cognitive electrophysiology [39]. Using decibel conversion, the change in power relative to baseline power is calculated using Equation (6):

(6) $$Normalized\;Power=10\cdot \log_{10}\left(\frac{activity\;power_{tf}}{baseline\;power_{f}}\right)$$

where t, and f indicate time and frequency points, respectively.

To be able to measure the baseline power, before conducting the experiment, the EEG signals for each subject were recorded while the participant was resting with her/his eyes open for 15 s, followed by another 15 s with closed eyes. These 30 s, including other 6 s for getting the subject ready, are considered as the baseline period. In addition to aforementioned benefits of baseline normalization, normalization makes it possible to remove the personal task-unrelated factors that might affect the results of the analysis.

### 3.5. Feature Selection

The total number of features included for each subject is 280. This high-dimensional feature vector makes the analysis more susceptible to overfitting. Thus, feature selection has to be performed before training a predictive model. There are many possible approaches for feature selection. A promising approach for feature selection is performed using transformation of the features (predictors) to fit the response variable (target) using regularized logistic regression [40]. In this approach, predictors are mapped to fit the target and the coefficients of the transformed predictors are used as a method for ranking the importance of them, where weak predictors tend to have small coefficients. In addition, to further enhance the performance of the feature selection process, a regularization term is added to the loss function to be minimized, which is E(X,Y), where X is the set of predictors and Y is the target. Thus, the objective function to be minimized is E(X,Y)+λ‖ω‖, where ‖⋅‖ denotes *l*1 or *l*2 regularizations and ω is the coefficients’ vector for the model.

### 3.6. Avoiding Overfitting

Another approach for speeding up learning and preventing over-fitting is to use regularization while training a model for classification. Different regularization approaches exist and can be effectively used with the learning algorithm to speed up learning and avoid over-fitting due to the small sample size and/or large number of features. For instance, as generally known, a multi-layer neural network (NN) consists of a large number of units (neurons) joined together in a pattern of connections. Feed-forward NNs allow signals to travel one way only, from input to output, where the input is, successively from one layer to another, multiplied by weights, summed up, then mapped to other values to produce the output by a pre-selected transfer function. The most well-known and widely used learning algorithm to estimate the values of the connecting weights is the back propagation (BP) algorithm. BP is a gradient descent (GD) algorithm that is usually used to minimize an objective function J(θ), where θ represents the model’s parameters. There are several implementations of GD algorithms that vary depending on the amount of data used in each step of parameters’ updates [41]. In batch GD (BGD), the complete training samples are used to calculate the updates of J(θ), whereas in stochastic GD (SGD), only a randomly-selected sample is used to update J(θ). Thus, J(θ) is updated using Equation (7):

(7) $$\theta =\theta -\eta \nabla_{\theta }J\left(\theta ,\;x^{i},\;y^{i}\right)$$

where η is the learning rate, and $x^{i},\;y^{i}$ are a training sample’s predictors and target, respectively for a classification problem. BGD produces better approximations of the objective function at the expense of time complexity. On the other hand, SGD handles the optimization problem with less time complexity at the expense of high variances of the minimized objective function. Thus, a combination between the two algorithms is usually used, namely mini-batch GD, where a small subset of the training samples is used for each iteration of the parameters’ updates.

It should be mentioned that η is used to regularize the GD algorithm by giving it large values in the beginning of the learning cycle to learn faster, then it is given small values to avoid oscillations around the local minima of the objective function. Another method to regularize learning is to use a momentum term γ. In this case, the rule used for minimizing J(θ) is given by Equation (8).

(8) $$\theta_{t}=\theta_{t-1}-\gamma V_{t-1}-\eta \nabla_{\theta }J\left(\theta_{t-1}\right)$$

where $V_{t-1}$ is the update vector used in the previous time step. To further optimize the previous equation, several optimization algorithms are introduced. For instance, the adaptive gradient algorithm (Adagrad), adaptive moment estimation algorithm (Adam), and Adamax (a variant of Adam based on infinity norm [41]) are among the used optimization algorithms that are based mainly on the adaptive variation of the learning rate and momentum values, for each element of θ, to speed up convergence and/or prevent the objective function from being trapped at local minima. Thus, the gradient of the objective function with respect to each parameter at time t, $\theta_{t,i}$ is used to find the gradient $g_{t,i}$ as given by Equation (9).

(9) $$g_{t,i}=\nabla_{\theta }J\left(\theta_{t,i}\right)$$

For instance, Adam [42] is based on storing the first and second momentums of the gradients as given by Equations (10) and (11), respectively.

(10) $$m_{t}=\frac{\beta_{1}m_{t-1}+\left(1-\beta_{1}\right)g_{t}}{1-\beta_{1}^{t}}$$

(11) $$v_{t}=\frac{\beta_{2}v_{t-1}+\left(1-\beta_{2}\right)g_{t}}{1-\beta_{2}^{t}}$$

where $m_{t}$ and $v_{t}$ are estimates of the first and second moment of the gradients, and $\beta_{1}$ and $\beta_{2}$ are the decaying rates for these two estimates, respectively. Usually, $l_{2}$ norm is used in the previous two equations to scale the gradients.

Finally, the parameters updating rule is given by Equation (12):

(12) $$\theta_{t+1}=\theta_{t}-\frac{\eta }{\sqrt{v_{t}}+\epsilon }m_{t}$$

where ϵ is a small value that prevents the division by zero.

In AdaMax, which is a slight modification of Adam, $l_{\infty }$ norm is used to update the gradients instead of $l_{2}$, which converges to a more stable solution with the default values of η=0.002, $\beta_{1}$ = 0.9, and $\beta_{2}$ = 0.999.

In multi-class classification problems, usually the objective function to be minimized represents the cross entropy (CE) [43], which is a measure of the difference between two probability distributions as given by Equation (13):

(13) $$CE=-\frac{1}{N}\overset{N}{\underset{i=1}{\sum }}\overset{C}{\underset{c=1}{\sum }}logP\left(y_{i}\epsilon C_{c}\right)$$

where *N* is the number of observations, *C* is the number of classes, and $y_{i}$ is the label (target) of the ith observation.

Although NNs are known to generalize well [44], they are usually susceptible to overfitting due to the large number of parameters that need to be optimized, especially when the number of observations is small and/or the features’ dimension is large. To further avoid over fitting, in addition to the regularized learning mentioned above, k-fold cross-validation is used. Moreover, the dropout approach [45] can be used. Dropout is a promising approach that was recently developed in deep learning to avoid deep networks from overfitting and improve their generalization ability. In this approach, during training, only randomly chosen percentage (p) of the neurons in the fully connected (dense) layers is included in the training, while the weights of the (1−p) remaining neurons are set to zero. In addition, in the testing phase, the weights of the neurons are modulated by p.

## 4. Results

### 4.1. Analysis of the Cognition Scores

**Figure 3:**
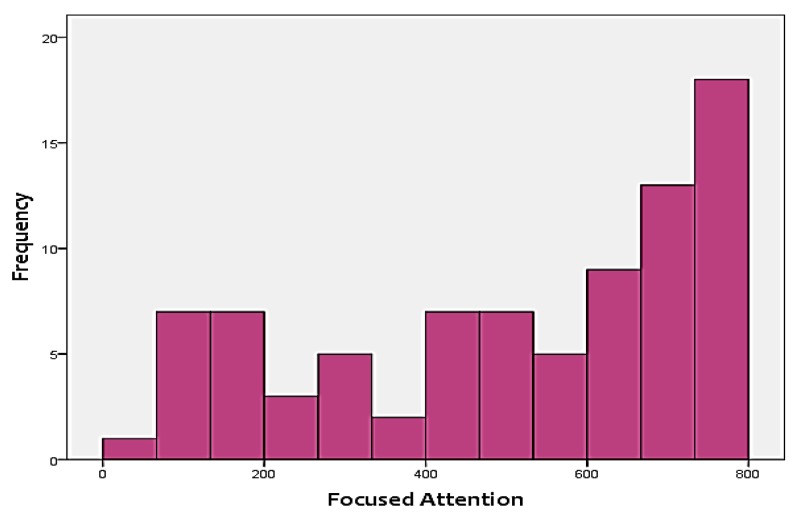
Distribution of focused attention (FA) test scores.

**Figure 4:**
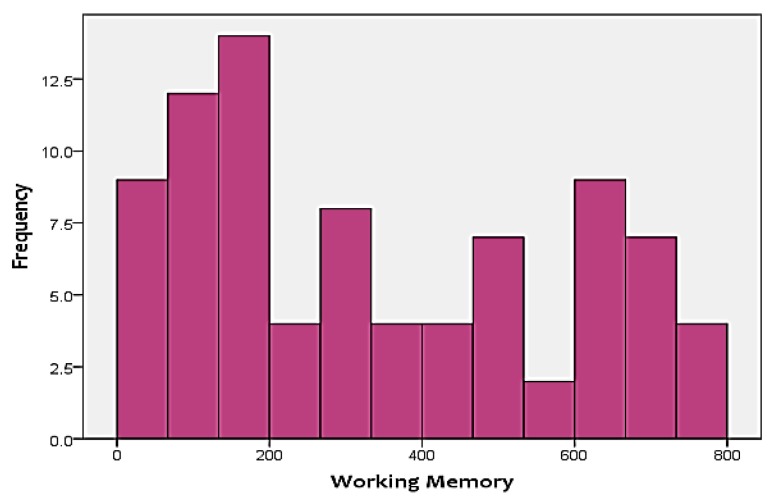
Distribution of working memory (WM) test scores.

As mentioned before, the two cognitive scores that were included in the analysis as the targets include FA and WM. Figure 3 and Figure 4 show the distribution of the two skills in the targets. As shown in the figures, none of the two targets follow a normal distribution. Instead, FA is positively-skewed, meanwhile, WM is negatively-skewed. This adds additional challenges to the analysis approach, since it has to be able to handle skewness of the output.

Furthermore, Spearman’s correlation coefficients between the targets were calculated. However, there was no significant correlation between them.

### 4.2. Feature Extraction

**Figure 5:**
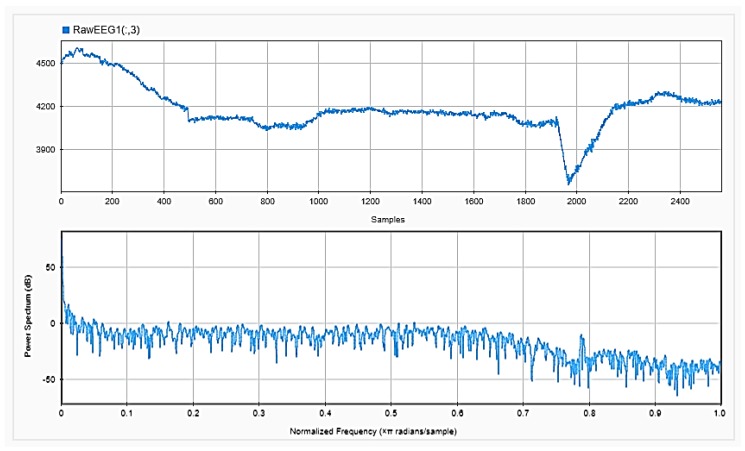
Raw EEG signals for one channel (**upper**), with its power spectral density (PSD) (**lower**).

**Figure 6:**
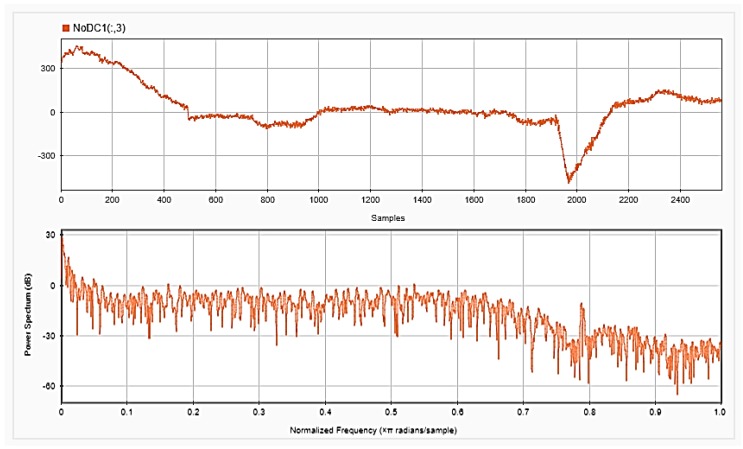
EEG signals for one channel after removing the DC offset in time (**upper**) and frequency (**lower**) domains.

**Figure 7:**
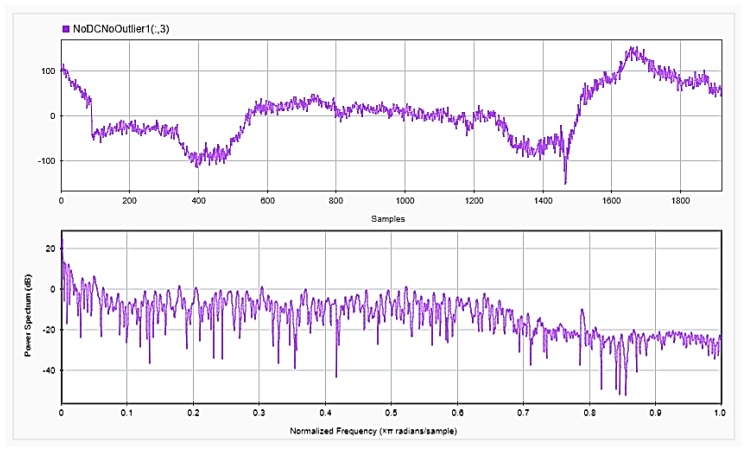
EEG signals for one channel after removing the DC offset and outliers in time (**upper**) and frequency (**lower**) domains.

**Figure 8:**
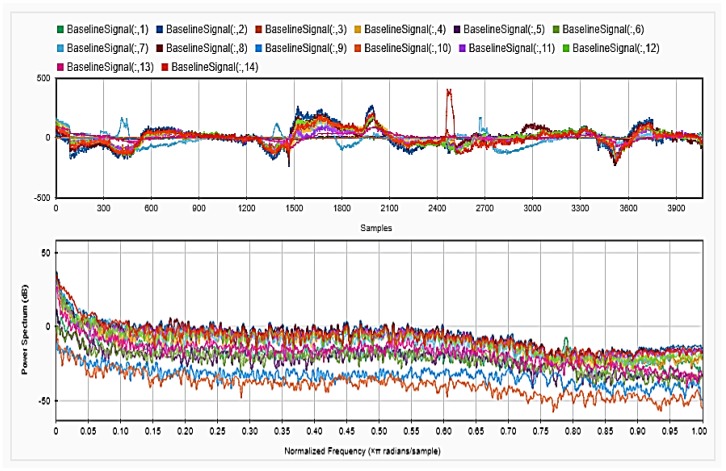
Baseline signal for the 14 channels in time (**upper**) and frequency (**lower**) domains.

**Figure 9:**
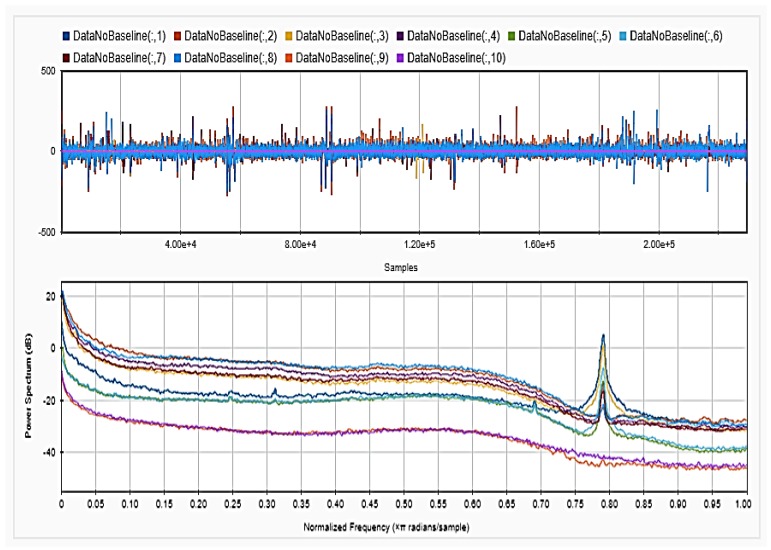
EEG signal after removing baseline period for one subject in time (**upper**) and frequency (**lower**) domains.

To be able to get information about the dynamics of the collected EEG data, features were extracted from the time as well as frequency domains. The analysis was repeated after each step of the implemented preprocessing. Figure 5, Figure 6 and Figure 7, respectively, show the first 15 s of the raw time series EEG for one channel and its PSD for one of the subjects, followed by those obtained for the same subject after removing the DC offset and the outliers. As shown in the figures, removing the DC has resulted in negative EEG values in the time domain. Furthermore, removing the outliers has resulted in a signal with smaller number of spikes in the time domain. Moreover, Figure 8 and Figure 9 show the baseline EEG signals for one of the subjects and the task-related EEG signals, respectively (in time and frequency domains). As shown in the figures, low frequencies have high power values as compared to high frequencies. In addition, when compared to the EEG signal before baseline normalization, it can be shown that is easier to differentiate between channels based on their power values, especially at small frequencies. It should be mentioned that the notch around 50 Hz (~0.8 Hz in terms of normalized frequencies) is mainly due to the low-pass filter with cutoff at 50 Hz that is used to avoid interference with the power line signals.

In total, 15 time-domain features were calculated for each channel, in addition to the extracted frequency-domain features, which include 5 power values for each of the 14-channel EEG signals; thus, in total 280 features were calculated for each subject.

**Figure 10:**
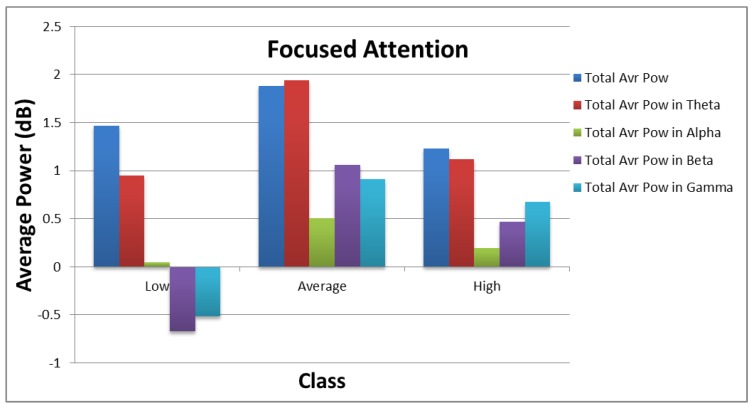
Distribution of average power values across different categories of focused attention.

**Figure 11:**
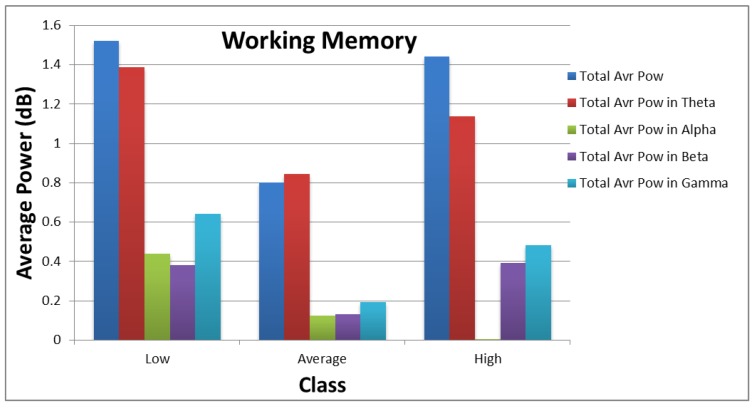
Distribution of average power values across different categories of working memory.

Figure 10 and Figure 11 show how the values of each subset of the frequency domain features vary across the different categories of each target. As shown in the figures, there are clear variations between the power values for each class of the targets.

### 4.3. Classification Results

To be able to correctly classify the collected EEG signals into one of the 3 classes (i.e., low, average and high) of each target, many classifiers were implemented using the python libraries numpy, Keras, scikit, and scipy. The trained classifiers include linear support vector (LinearSVC), random forest (RF), support vector classifier with radial basis function (SVC), k-nearest neighbor (KNN), neural networks (NN), decision trees (DT), Gaussian process (GP), boosted and bagged ensembles (tree and KNN-based), and random-under sampling boosted trees. However, with the skewness of the data and the relatively-small number of observations in comparison to the features’ dimension, it was expected that only a few of the classifiers would be able to yield good results, even after hyper-parameter optimization. It should be mentioned also that in all of the performed experiments, the hyper-parameters of the implemented classifiers were tuned using the grid search method in scikit-learn library. The calssifiers aforementioned have been tested using the time and frequency-domain features computed from the (1) raw data, (2) data with DC component removed (*NoDC*) and (3) data with the DC and outliers removed (*NoOutliers*).

To avoid overfitting, meta-transformation feature selection was applied using a logistic regression classifier with k-fold cross validation (k=10) and the mean score is obtained. Also, regulization with different optimizitation techniques were investigated. Moreover, dropout was used when NN is employed as the classifier.

In the following subsections, the performances of the implemented classifiers for the three data sets (raw data, *NoDC*, *NoOutliers*) using only time-domain features, only frquency-domain features, and combined set of features, will be presented.

#### 4.3.1. Classification Results Using Only Time-Domain Features

##### Classification of Focused Attention

**Figure 12:**
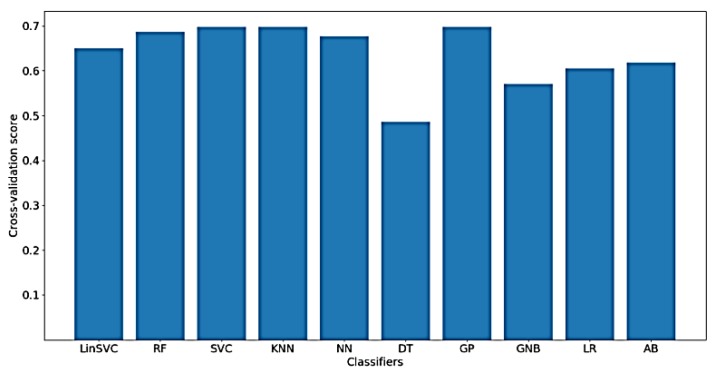
Performance of implemented classifiers for the classification of FA scores using raw data and only time-domain features.

Figure 12 shows the classification accuracy for the implemented classifiers on raw data. As shown in the figure, the highest obtained classification accuracy is about 70%. The classifiers that yielded the best accuracy include SVC, KNN and GP. It should be mentioned that, after feature selection, only 70 out of the 182 time domain features were selected. In addition, the selected features mostly belong to shapiro-wilk test for normality and Hjorth mobility and complexity values.

**Figure 13:**
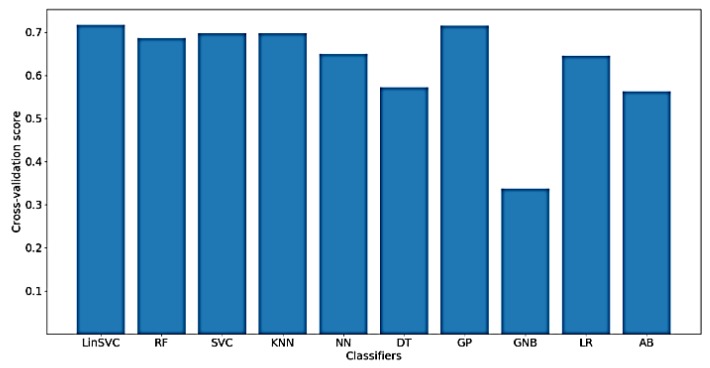
Performance of implemented classifiers for the classification of FA scores using no DC component (*NoDC*) data and only time-domain features.

Furthermore, Figure 13 shows the classification accuracy for the implemented classifiers on *NoDC*. As shown in the figure, the highest obtained classification accuracy is also about 70%. This is expected, since removing the DC component should not affect the time domain characteristics of the signal. Moreover, the classifiers that yielded the best accuracy include LinearSVC and GP. After feature selection, only 78 out of 182 time domain features were selected.

**Figure 14:**
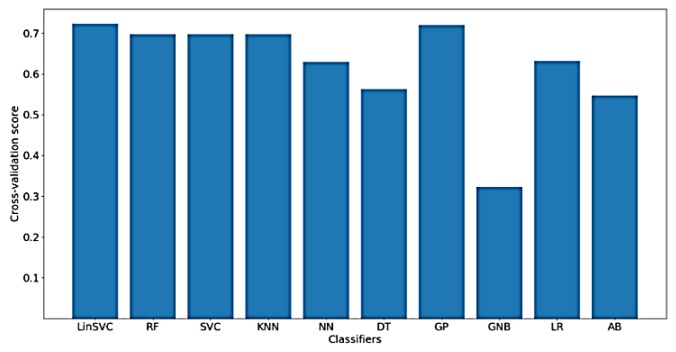
Performance of implemented classifiers for the classification of FA scores using *NoOutliers* data and only time-domain features.

In addition, Figure 14 shows the classification accuracy for the implemented classifiers on *NoOutliers* data. As shown in the figure, the highest obtained classification accuracy is also about 70%. This may be explained by the low ratio of removed outliers from each signal, hence approximately the same performance measures are obtained when *NoOutlier* data are used in comparison with the raw and *NoDc* data. Another implication might be that there is no information related to the FA in the time-domain charactersitics of the outliers. As shown in the figure, the classifiers that yielded the best accuracy include LinearSVC and GP. After feature selection, only 76 out of 182 time-domain features were selected.

##### Classification of Working Memory (WM)

**Figure 15:**
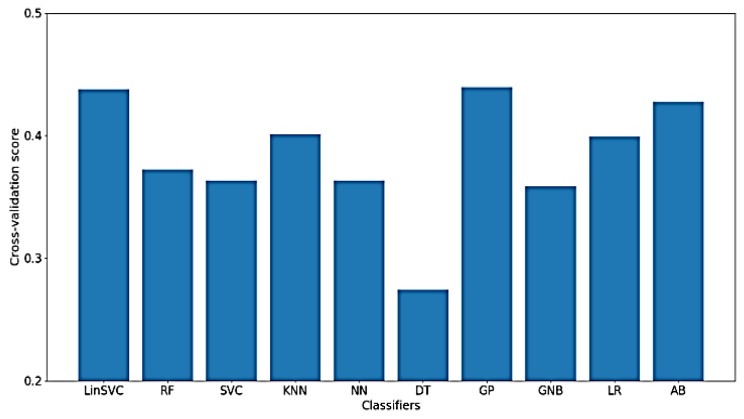
Performance of implemented classifiers for the classification of WM scores using raw data and only time-domain features.

As for the classification of WM, much worse results were obtained using only time-domain features, which means that the characteristics of the signal related to WM cannot be extracted from the time-domain only. Figure 15 presents the classification accuracy for the implemented classifiers on raw data using WM as the target. As shown in the figure, the obtained classification accuracies are below 50%, where there are only 77 out of 182 time-domain features.

**Figure 16:**
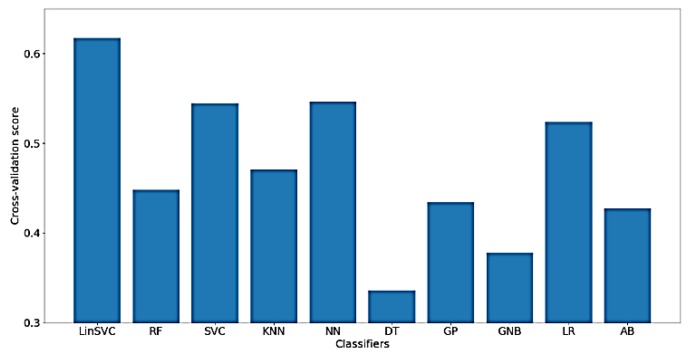
Performance of selected classifiers for the classification of WM scores using *NoDC* data and only time-domain features.

Furthermore, Figure 16 presents the classification accuracy of WM scores for the implemented classifiers using *NoDC*. As shown in the figure, the highest obtained classification accuracy is, surprisingly, increased to about 63% using LinearSVC. Also, after feature selection, only 83 out of 182 time-domain features were selected.

**Figure 17:**
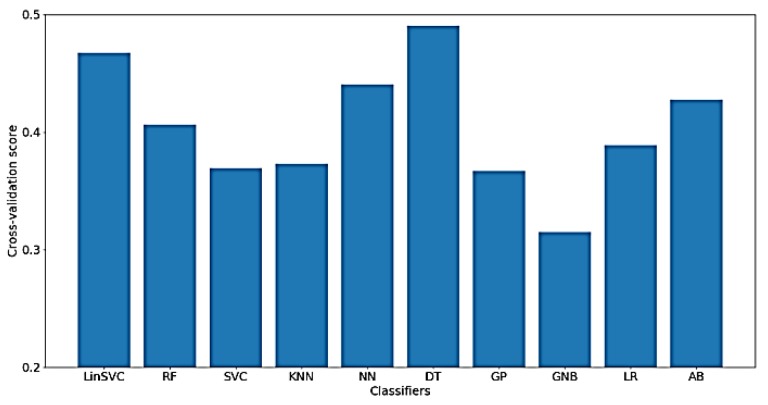
Performance of selected classifiers for the classification of WM scores using *NoOutlier* data and only time-domain features.

In addition, Figure 17 presents the classification accuracy of WM for the implemented classifiers on *NoOutliers* data. As shown in the figure, the highest obtained classification accuracy is below 50%, which is obtained using only 97 features.

It should be mentioned that, in general, the classification results using the *NoDC* data were higher than those obtained when both the raw or *NoOutliers* data were used. This implies that the outliers that were detected in the EEG signals might contain some information about the predicted cognitive skills and hence, should not be removed. Therefore, in the following subsections, the obtained results on only *NoDC* data will be reported.

#### 4.3.2. Classification Results Using Only Frequency-Domain Features

When only the frequency-domain features are used, approximately similar results were obtained. To provide some information about the hyper-parameters of the used models, the results are listed in a table. Table 1 summarizes the classification results using only the 70 frequency-domain features. As can be shown in the table, KNN gave the highest classification scores of 70% and 51% for FA and WM, respectively. It is worth mentioning that the selected features mostly include the power in the theta band for the different channels.

#### 4.3.3. Classification Results Using Combined Features

The two features’ sets were combined, which resulted in a total number of statistical and frequency domain features of 280. This number was then reduced using the same feature selection approach that was used before. The best obtained results are summarized in Table 2, together with some information on the important hyper-parameters that yielded best results for the used classifiers. As can be shown in the table, combining the time and frequency-domain features yielded the best results, which indicate that both the time and frequency domains have information about the detected cognitive skills that need to be used in encoding them. Moreover, the best obtained accuracy for FA was 84% using a two-layer NN, meanwhile, linear support vector classifier yielded the best accuracy (about 81%) for the detection of WM. Other classifiers, however, were not able to provide good performance on the used data set. It is worth mentioning that the selected features in the best models include mostly power band and Hjorth mobility and complexity values.

## 5. Conclusions and Future Work

In this paper, an approach for the prediction of focused attention and working memory using EEG is proposed. EEG signals were recorded while the subjects undertook a cognitive test that stimulated these cognitive skills. The collected EEG signals were analyzed in the time and frequency domains to extract a set of 280 features, which were then used to train different classifiers. The built models were able to detect the three levels of FA and WM with good accuracy, which indicates the suitability of the proposed approach to predict the three levels (i.e., low, average, and high) of focused attention and working memory of learners using a wireless 14-channel EEG headset. In comparison with similar works, the proposed models provide generalizable and consistent results since they were obtained using a relatively-large sample size. For example in [5,46], a sample size of 24 and 4 participants were used, respectively, where cognitive states were classified into only two classes; attentive and non-attentive. In addition, the best obtained classification accuracies were 77% and 83%, respectively, using SVM binary classifiers. A comparative analysis with similar work is presented in Table 3. It should be mentioned that only approaches with machine learning-based models are included in the table; studies with statistical designs are not added.

As a future work, to avoid feature extraction and selection, using the raw EEG for the detection of the levels of FA and WM will be examined. For this purpose, deep-learning approaches such as convolutional and recurrent neural networks can be used. As a limitation of the study, it should be mentioned that a few of the subjects reported difficulty in recognizing the meanings of some English words that were included in the cognitive test, which should be considered in the future as it might have affected the analysis. Another limitation of the study is that the data collection was performed in a laboratory-like setting, not in a real classroom environment. Therefore, the factors that might affect the results when the experiment is conducted in a more ecological setting need to be considered.

## Figures and Tables

**Table 1:** Summary of the average scores of stratified 10-fold cross validation for the two cognitive skills using only frequency-domain features. Highest scores are shown in bold.

Classifier	Parameters	Focused Attention	Working Memory
Linear Support Vector (LinSVC)	Uses l2-norm penalty	0.69	0.42
Random Forest (RF)	Number of trees = 200	0.66	0.48
Support Vector Classifier (SVC)	Uses a radial basis function kernel	**0.7**	0.47
K-Nearest Neigbor (KNN)	K = 15	**0.7**	**0.5**
Neural Network (NN)	Two hidden layers with 40 nodes each, logistic activation and regularization parameter of 0.001.	0.68	0.37
NN with Dropout layers	Two hidden dense layers of 271 and 180 nodes followed by a 25% dropout layer, followed by a hidden dense layer of 280 nodes and a 25% dropout layer, All layers have sigmoid activation with Adamax optimization.	0.66	0.39
Decision Tree (DT)	Nodes are expanded until all leaves are pure or until all leaves contain less than the minimum number of samples required to split an internal node, which is 2. The minimum number of samples required to be at a leaf node is 1.	0.53	0.35
Gaussian Process (GP)	The kernel is radial-basis function	0.65	0.35
Gaussian Naive Bayes (GNB)		0.51	0.48
Logistic Regression (LR)	The penalty is L1 norm. Inverse of regularization strength is 1.	**0.7**	0.43
Ada Boost (AB)	DT as base estimator.	**0.7**	0.42

**Table 2:** Summary of the average scores of stratified 10-fold cross validation for the two cognitive skills using statistical and frequency domain features. Highest scores are shown in bold.

Classifier Name	Parameters	Focused Attention	Working Memory
Linear Support Vector (LinSVC)	Uses l2-norm penalty	0.80	**0.815**
Random Forest (RF)	Number of trees = 200	0.70	0.55
Support Vector Classifier (SVC)	Uses a radial basis function kernel	0.69	0.58
K-Nearest Neigbor (KNN)	K = 15	0.69	0.46
Neural Network (NN)	Two hidden layers with 40 nodes each, logistic activation and regularization parameter of 0.001.	**0.84**	0.811
Neural Network with Dropout layers	Two hidden dense layers of 271 and 180 nodes followed by a 25% dropout layer, followed by a hidden dense layer of 280 nodes and a 25% dropout layer, All layers have sigmoid activation, with AdaMax optimization.	0.82	0.74
Decision Tree (DT)	Nodes are expanded until all leaves are pure or until all leaves contain less than the minimum number of samples required to split an internal node, which is 2. The minimum number of samples required to be at a leaf node is 1.	0.59	0.43
Gaussian Process (GP)	The kernel is radial-basis function	0.68	0.43
Gaussian Naive Bayes (GNB)		0.45	0.40
Logistic Regression (LR)	The penalty is L1 norm. Inverse of regularization strength is 1.	0.62	0.64
Ada Boost (AB)	DT as base estimator.	0.69	0.42

**Table 3:** The proposed approach in comparison with related work.

Ref.	Detected Cognitive State(s)	Sample Size	Used Model(s)	Performance
[5]	Attention	24	Support Vector Machines (SVM)	76.8%
[8]	Four Attention states	4	NN	56.5–79.75%
[9]	Discrimination between working memory and recognition	10	SVM	79%
[19]	Four cognitive states related to an ambulatory subject	A total of 6000 data points taken from 1 subject	Ensemble Classifier	80%
[21]	Cognitive states related to viewing 4 images	26	Convolutional NN	79.9%
Proposed Approach	Three different levels (low, average, high) for the working memory and focused attention	86	NN and SVC	84% for WM and 81% for FA
